# A Life without Hunger: The Ups (and Downs) to Modulating Melanocortin-3 Receptor Signaling

**DOI:** 10.3389/fnins.2017.00128

**Published:** 2017-03-16

**Authors:** Andrew A. Butler, Clemence Girardet, Maria Mavrikaki, James L. Trevaskis, Heather Macarthur, Daniel L. Marks, Susan A. Farr

**Affiliations:** ^1^Department of Pharmacology and Physiology, Saint Louis University School of MedicineSt. Louis, MO, USA; ^2^In vivo Pharmacology, Cardiovascular and Metabolic Disease, MedimmuneGaithersburg, MD, USA; ^3^Papé Family Pediatric Research Institute, Oregon Health and Science UniversityPortland, OR, USA; ^4^Department of Internal Medicine, Division of Geriatrics, Saint Louis University School of MedicineSt. Louis, MO, USA; ^5^VA Medical CenterSt. Louis, MO, USA

**Keywords:** obesity, diabetes, appetite, neuropeptide, hypothalamus, limbic system, homeostasis, metabolism

## Abstract

Melanocortin neurons conserve body mass in hyper- or hypo-caloric conditions by conveying signals from nutrient sensors into areas of the brain governing appetite and metabolism. In mice, melanocortin-3 receptor (MC3R) deletion alters nutrient partitioning independently of hyperphagia, promoting accumulation of fat over muscle mass. Enhanced rhythms in insulin and insulin-responsive metabolic genes during hypocaloric feeding suggest partial insulin resistance and enhanced lipogenesis. However, exactly where and how MC3Rs affect metabolic control to alter nutrient partitioning is not known. The behavioral phenotypes exhibited by MC3R-deficient mice suggest a contextual role in appetite control. The impact of MC3R-deficiency on feeding behavior when food is freely available is minor. However, homeostatic responses to hypocaloric conditioning involving increased expression of appetite-stimulating (orexigenic) neuropeptides, binge-feeding, food anticipatory activity (FAA), entrainment to nutrient availability and enhanced feeding-related motivational responses are compromised with MC3R-deficiency. Rescuing *Mc3r* transcription in hypothalamic and limbic neurons improves appetitive responses during hypocaloric conditioning while having minor effects on nutrient partitioning, suggesting orexigenic functions. Rescuing hypothalamic MC3Rs also restores responses of fasting-responsive hypothalamic orexigenic neurons in hypocaloric conditions, suggesting actions that sensitize fasting-responsive neurons to signals from nutrient sensors. MC3R signaling in ventromedial hypothalamic SF1(+ve) neurons improves metabolic control, but does not restore appetitive responses or nutrient partitioning. In summary, desensitization of fasting-responsive orexigenic neurons may underlie attenuated appetitive responses of MC3R-deficient mice in hypocaloric situations. Further studies are needed to identify the specific location(s) of MC3Rs controlling appetitive responses and partitioning of nutrients between fat and lean tissues.

Obesity is often attributed to a combination of genetic susceptibility and imbalances between energy intake and expenditure (Hill et al., 2012; Speakman and O'Rahilly, 2012). The problem facing modern societies is that obesity is now common: two-thirds of the population in the United States are overweight or obese (Lewis et al., 2009). Obesity increases risk of cardiometabolic disease and some cancers, reducing quality and duration of life (Lewis et al., 2009). Determining why some become obese and some do not is fundamental to solving and perhaps reversing current obesity trends. MC3Rs are a component of a canonical hypothalamic neural network regulating body mass and substrate partitioning between adipose and lean tissues (Girardet and Butler, 2014). While not widely considered a target for obesity treatment, here we discuss recent studies suggesting the importance of MC3Rs in appetite and metabolic control.

## An overview of the central nervous melanocortin system

At the core of central nervous melanocortin system are two neuronal populations sending projections throughout the brain from soma in the hypothalamic arcuate nucleus (ARC). These neurons integrate humoral cues of metabolic condition (insulin, acyl-ghrelin, leptin, glucagon-like peptide-1, glucocorticoids, interleukins and estrogen) (Mauvais-Jarvis et al., 2013; Gautron et al., 2015), metabolites such as glucose (Ibrahim et al., 2003; Parton et al., 2007), and inputs from neurons releasing serotonin (Burke and Heisler, 2015), glutamate (Krashes et al., 2014), orexin (van den Top et al., 2004; Morello et al., 2016), and cannabinoids (Koch et al., 2015; Morello et al., 2016).

**Figure 1:**
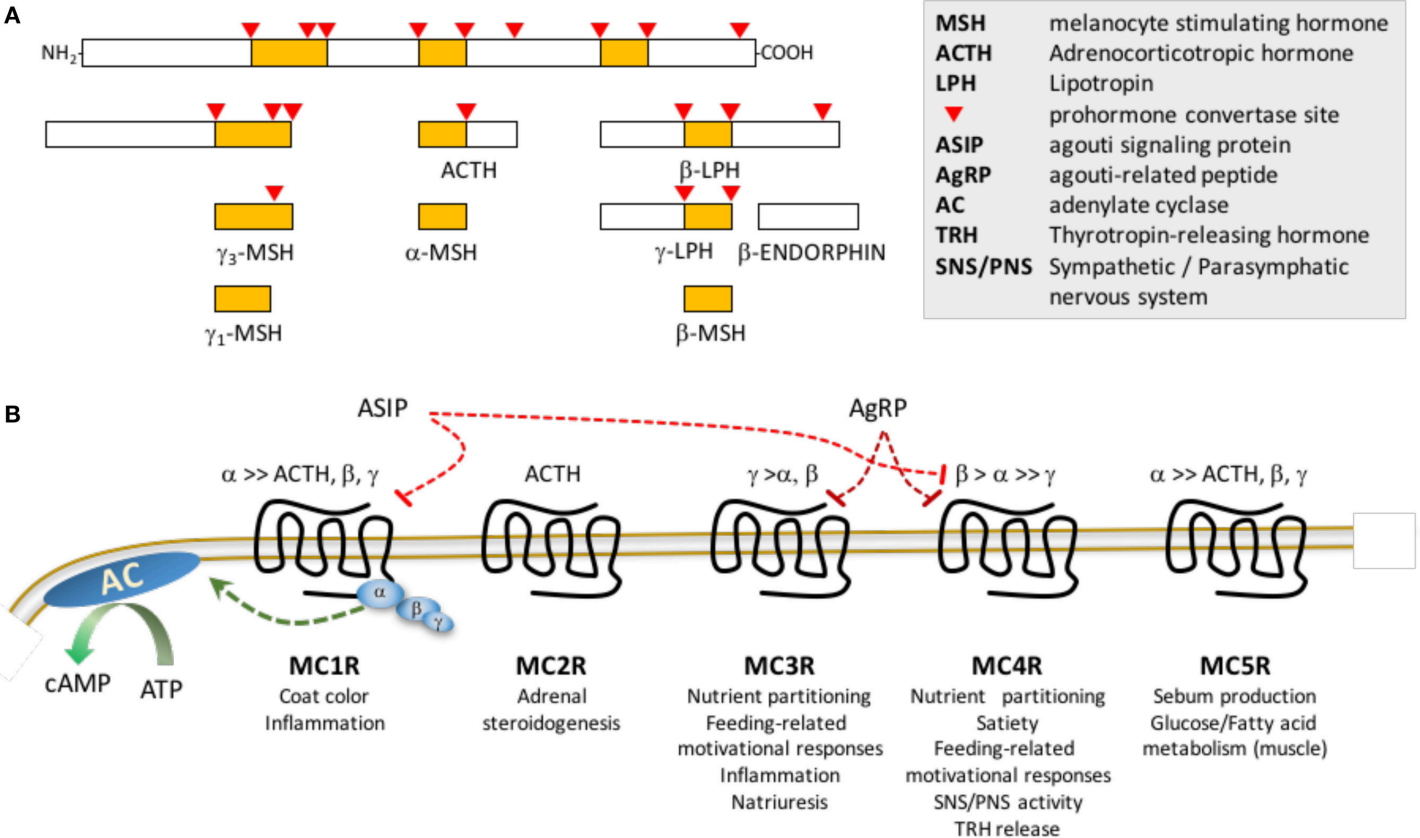
**Post-translational processing of POMC (A)** and melanocortin receptor pharmacology **(B)**. **(A)** The propeptide proopiomelanocortin (POMC) is post-translationally modified by serine proteases propeptide (also known as prohormone) convertases 1/3 and 2. **(B)** MSH peptides and ACTH peptides activated following release from POMC differ in affinity for the 5 members of the melanocortin receptor family: γ–MSH exhibits preferential affinity for MC3Rs; ACTH is the only agonist for MC2Rs; ASIP exhibits high affinity for MC1R and MC4R, while AgRP is a selective antagonist for MC3R and MC4R. Note that this is a simplified schematic, and does not show the melanocortin receptor accessory proteins (MRAP1, MRAP2) that associate with the melanocortin receptors to modify receptor activity or coupling to β–arrestins which mediates receptor internalization and activation of intracellular signaling cascades. Receptor binding of the MSH results in activation of the stimulatory subunit of trimeric G protein receptor complex (α,β,γ) for all members of the family, resulting in increased adenylate cyclase (AC) activity and accumulation of cAMP. Agouti signaling peptide (ASIP) and agouti-related peptide (AgRP) were initially described as antagonists, however they may have biased agonist properties, activating receptor coupling to other G protein complexes. Lists of physiological processes are shown below each receptor.

GABA-ergic neurons co-expressing orexigenic neuropeptides agouti-related peptide (AgRP) and neuropeptide Y (NPY) are activated upon fasting (Hahn et al., 1998; Betley et al., 2015). Activation of NPY/AgRP/GABA (NAG) neurons rapidly induces feeding and learned instrumental actions to obtain food (Aponte et al., 2011; Krashes et al., 2011). In contrast, ablation causes anorexia and impairs adaptation to hypocaloric conditioning (Bewick et al., 2005; Luquet et al., 2005; Tan et al., 2014). Another population of ARC neurons express proopiomelanocortin (POMC), a propeptide converted to β–endorphin (an endogenous opioid) and melanocortins (α–, β– and γ–MSH and ACTH) (Figure 1A; Cone, 2006). Activation of ARC POMC neurons in mice inhibits feeding behavior, albeit over longer time frames compared to NAG neurons (Zhan et al., 2013). In contrast, ablating POMC neurons or suppressing ARC *Pomc* expression causes hyperphagic obesity syndromes (Smart et al., 2006; Bumaschny et al., 2012; Zhan et al., 2013). Activation of small population of POMC neurons in the nucleus of the solitary tract of the hindbrain rapidly inhibits feeding, however their ablation does not produce obesity (Zhan et al., 2013).

### Cloning of the melanocortin receptors

Physiological responses to melanocortin ligands are mediated by five receptors (MC1R-MC5R) (Cortés et al., 2014). Melanocortin receptor pharmacology is complex, with two antagonists/inverse agonists (AgRP and agouti signaling peptide) and MSH ligands that exhibit varying degrees of receptor specificity (Figure 1B; Cone et al., 1996). Other ligands and cell-surface proteins have been identified that regulate melanocortin signaling (e.g., melanocortin receptor accessory proteins 1 and 2, mahogany, mahoganoid, attractin-like protein, syndecans, ion channels and defensins) (Kaelin et al., 2008; Nix et al., 2013, 2015; Anderson et al., 2016).

Melanocortin regulation of energy balance is mediated by two receptors expressed in the central nervous system. *Mc3r* and *Mc4r* mRNA are expressed in overlapping and distinct brain regions linked to appetite and metabolic control (Roselli-Rehfuss et al., 1993; Mountjoy et al., 1994; Kishi et al., 2003; Liu et al., 2003; Lippert et al., 2014; Mavrikaki et al., 2016). *Mc3r* expression is concentrated in hypothalamic and limbic structures, with dense expression in the ARC, ventromedial hypothalamus (VMH), ventral tegmental area (VTA), and medial habenula (MHb) (Roselli-Rehfuss et al., 1993; Cone, 2005; Lippert et al., 2014; Mavrikaki et al., 2016). Initial observations of expression of both receptors in areas of the rodent brain linked to appetite control (Roselli-Rehfuss et al., 1993; Mountjoy et al., 1994), and stimulation of feeding by melanocortin antagonists administered centrally (Fan et al., 1997), were crucial early steps in revealing the physiological significance of the central nervous melanocortin system. Chronic intracerebroventricular infusion of AgRP, an MC3R/MC4R antagonist/inverse agonist (Ollmann et al., 1997; Shutter et al., 1997), causes a hyperphagic obesity syndrome (Small et al., 2001). The central nervous melanocortin system is thus viewed as a promising target for developing obesity therapies. The first trials of melanocortin agonists for treating obesity failed due to cardiovascular responses (Greenfield, 2011). However, a recent trial investigating RM-493, a small peptide MC3R/MC4R agonist shown to have MC4R-dependent effects on food intake and body weight (Kumar et al., 2009), produced promising outcomes. In humans, RM-493 increased resting energy expenditure and reduced the respiratory quotient (RQ), suggesting enhanced fat oxidation (Chen et al., 2015). In obese non-human primates, administration of RM-493 resulted in weight loss with a transient suppression of food intake, increased total energy expenditure and improvements in insulin resistance and cardiovascular function (Kievit et al., 2013). Importantly, adverse cardiovascular responses that led to the discontinuation of earlier compounds were not evident.

In the absence of selective melanocortin receptor ligands, targeted deletion of the melanocortin receptors provided important information concerning the functional specificity of neural melanocortin receptors. MC3Rs are not required for suppression of food intake in response to MSH analogs (Marsh et al., 1999; Chen et al., 2000a,b; Kumar et al., 2009), and for appetite control during exposure to palatable high-fat/high sucrose diets (Butler et al., 2000, 2001; Albarado et al., 2004; Sutton et al., 2006; Srisai et al., 2011). Unlike MC4Rs, MC3Rs are not required for appetitive and metabolic responses to serotoninergic compounds (Heisler et al., 2002, 2006; Zhou et al., 2007). Deletion of the gene encoding either MC3R or MC4R causes obesity in mice, with both affecting partitioning of nutrients between adipose and non-adipose tissues (Huszar et al., 1997; Butler et al., 2000; Chen et al., 2000a). The two receptors were originally considered to function independently, as *Mc3r;Mc4r* double knockouts exhibit an additive obese phenotype (Chen et al., 2000a). As discussed later in this review, our data suggest MC3Rs may regulate MC4R activity by altering the response of “1st order” neurons releasing the endogenous ligands to signals of metabolic state.

Genetic screens of obese populations confirmed the importance of normal melanocortin receptor function in the defense of body weight from early childhood. Missense mutations in the *POMC* and *MC4R* genes are associated with severe hyperphagic obesity syndromes that manifests within the first 1–2 years of life (Farooqi and O'Rahilly, 2008). The central nervous melanocortin system responds to environmental cues through epigenetic modifications that have long-lasting effects on expression of genes promoting lean phenotypes (Benite-Ribeiro et al., 2016; Kühnen et al., 2016). Methylation in a variably methylated region (VMR) of the *POMC* gene allele is associated with altered body mass in humans (Kühnen et al., 2016). Methylation of this region is sensitive to metabolic conditions *in utero*, and to paternal methylation patterns. Altered *POMC* expression as a consequence of developmental conditions could therefore contribute to obesity later in life. While the evidence for direct causality is less clear, *MC3R* haploinsufficiency is linked to increased risk of childhood obesity (Feng et al., 2005; Tao, 2010; Lee, 2012; Lee et al., 2016).

## Is there a role for MC3Rs in appetite regulation?

Expression of MC3Rs in limbic and hypothalamic structures suggests functions related to controlling complex behaviors, including appetite (Roselli-Rehfuss et al., 1993; Lippert et al., 2014; Mavrikaki et al., 2016). However, as discussed above characterization of feeding behavior in *Mc3r* knockout (−/−) mice on mixed or congenic (C57BL/6J) backgrounds has been inconclusive (Butler et al., 2000; Chen et al., 2000a; Butler, 2006; Sutton et al., 2006; Ellacott et al., 2007; Begriche et al., 2011a).

Recent results from a recent experiment in mice with “humanized” MC3Rs may suggest a role in appetite control (Lee et al., 2016). Risk of childhood obesity is increased in homozygous carriers of two *MC3R* sequence variants (C17A+G241A) that reduce receptor binding and maximal cAMP accumulation in cell-based assays (Feng et al., 2005). Mice homozygous for the mutant *hMC3R* containing the double mutation (*MC3R*^*hDM*/*hDM*^) exhibit reduced musculoskeletal mass and increased adiposity when compared to mice inheriting “wild type” hMC3Rs (*MC3R*^*hWT*/*hWT*^) (Lee et al., 2016). *MC3R*^*hDM*/*hDM*^ mice are also hyperphagic; while the difference is small (1–2 kcal/mouse/day), over time this could produce significant changes in adiposity (Butler and Kozak, 2010). However, hyperphagia does not explain the nutrient-partitioning defect reducing musculoskeletal growth, which has been postulated to result from a mild Cushingoid phenotype (Renquist et al., 2012).

How the feeding phenotype of *MC3R*^*hDM*/*hDM*^ mice compares to outcomes from other studies using *Mc3r*-deficient mice is unclear. While classical gene targeting techniques result in complete loss of MC3R signaling, some signaling is presumably retained in *MC3R*^*hDM*/*hDM*^ mice. Information on the impact of the (C17A+G241A) mutation on second messenger signaling thus far has been limited to measuring cAMP accumulation in the presence of the synthetic analog [Nle^4^, D-Phe^7^]-α-MSH. Information on how the mutation alter other signaling mechanisms and responses to other ligands such as AgRP are not available, but could be relevant given that physiological responses to centrally administered melanocortin agonists involve distinct G protein signaling mechanisms (Li et al., 2016).

### MC3R role in appetite regulation is context-dependent and exposed in hypocaloric conditions

Overall, the lack of conclusive evidence supporting a role for MC3Rs in appetite control in *ad libitum* fed situations, combined with comparatively modest changes in body mass (Butler et al., 2000, 2001; Chen et al., 2000a), explains why many laboratories overlooked neural MC3Rs. Evaluating behavioral and/or metabolic responses of mice to environmental challenges can be informative when investigating the functions of genes involved in behavior and metabolism. For example, cold stress is often used to assess mobilization of energy reserves and futile cycles to maintain body temperature (Kozak and Anunciado-Koza, 2008). Another example is transitioning between chows and obesogenic diets to assess behavioral and metabolic control (Collins et al., 2004). *Mc3r*-deficient mice tolerate cold and control appetite when challenged with palatable diets (Butler et al., 2000, 2001; Chen et al., 2000a; Sutton et al., 2006; Ellacott et al., 2007). However, a behavioral phenotype is observed in *Mc3r*−/− mice subjected to hypocaloric restricted feeding protocols to assess motivational responses anticipating food presentation (Sutton et al., 2008; Begriche et al., 2011a,b, 2012; Girardet et al., 2014a, 2017). These outcomes suggest that MC3Rs play a role in mediating appetite responses to situations of nutrient scarcity.

Mice provided unrestricted access to a running wheel exhibit food anticipatory activity (FAA) when subjected to a hypocaloric diet (70–75% of habitual intake) presented at 24 h intervals (Mistlberger, 2011). FAA involves a progressive rise in activity preceding food access, and has been suggested to involve a circadian oscillator (“food-entrainable oscillator,” or FEO) that is independent of the light-entrained master clock. FAA is attenuated in *Mc3r*−/− mice housed in a 12 h light:dark setting (Sutton et al., 2008); the same study reported that *Mc3r*−/− mice failed to increase wakefulness in anticipation of food presentation. Entrainment to food presentation is also attenuated, but not completely inhibited, when FAA is assessed in constant dark (Begriche et al., 2011b). Based on the weakened anticipatory responses observed during restricted feeding, MC3Rs may act as a modulator of the inputs (or outputs) of FEOs (Mistlberger, 2011). Entrainment to food availability is thought to involve coordinated responses of FEO distributed throught the body (Mohawk et al., 2012). However, it is no clear how MC3Rs exert regulatory control over rhythms in FEO activity.

A recent paper from Roger Cone's laboratory suggested another interpretation of the FAA phenotype associated with loss of MC3R. Renquist et al. reported that the fasting responses of NAG neurons are not observed in *Mc3r*−/− mice (Renquist et al., 2012). We subsequently reported increased hypothalamic *AgRP* and *Npy* expression in the hypocaloric conditions used to induce FAA is also not observed in *Mc3r-*deficient mice (Girardet et al., 2014a, 2017). Collectively, these results suggest activation of NAG neurons by signals of negative energy balance contributes to the expression of FAA. Adult mice lacking NAG neurons adapt poorly to a hypocaloric feeding protocol used to induce FAA (Tan et al., 2014). FAA involves increased food seeking and motivational responses to seek food (Aponte et al., 2011; Krashes et al., 2011). Similar responses occur upon activation of NAG neurons (Aponte et al., 2011; Krashes et al., 2011), although another interpretation is that activation of NAG neurons delivers a “negative valence” signal (Betley et al., 2015) causing avoidance of situations associated with a painful experience (hunger).

*Mc3r-*deficient mice also exhibit attenuated appetitive responses to hypocaloric conditioning. Wild-type mice subjected to hypocaloric feeding protocols exhibit binge-feeding behavior, reducing meal frequency and increasing meal size to consume most of the food within 1 h of presentation (Bruss et al., 2010; Begriche et al., 2012; Girardet et al., 2017). This behavioral adaptation is attenuated in *Mc3r*−*/*− mice: food intake in the 1 h following presentation is markedly reduced with no compensation later in the feeding cycle and changes in meal structure (fewer, larger meals) are also attenuated (Begriche et al., 2012; Girardet et al., 2017). Motivation to self-administer food-rewards during hypocaloric conditions is also attenuated in *Mc3r*-deficient mice (Mavrikaki et al., 2016). However, self-administration is normal in *Mc3r*-deficient mice in *ad libitum* feeding conditions and increased motivation to self-administer more palatable sucrose diets is retained (Mavrikaki et al., 2016). The behavioral phenotype associated with MC3R-deficiency is therefore contextual and dependent on energy balance. *Mc3r*-deficient mice may not experience the “pain” of hunger, and are not be motivated to avoid unpleasant experiences associated with nutrient insufficiency.

These observations also suggest a new and perhaps simpler interpretation of the phenomenon observed in *Mc3r*−*/*− mice during restricted feeding. In the absence of MC3Rs, NAG neurons are desensitized to internal cues of metabolic state provided by hormones and metabolites, the release of which follows patterns that are sensitive to food consumption (Tschop et al., 2006). This model also explains why the release of other neuropeptides and neurotransmitters from NAG neurons does not compensate for the absence of MC3Rs. The rapid stimulation of feeding behavior following activation of NAG neurons requires the release of GABA or NPY from NAG neurons, while release of AgRP elicits a delayed yet prolonged increase in feeding behavior that is dependent on MC4Rs (Krashes et al., 2013).

### MC3Rs in hypothalamic and limbic structures promote appetitive responses to hypocaloric conditions

We developed the LoxTB*Mc3r* mouse, allowing us to reactivate of *Mc3r* transcription using Cre transgenics, inserting a “lox-stop-lox” sequence in the 5′UTR (Begriche et al., 2011a). The response of NAG neurons to hypocaloric conditioning is restored in LoxTB*Mc3r* mice in which hypothalamic expression was rescued using Nkx2.1-Cre (Girardet et al., 2017). This study also observed that restoring FAA in LoxTB*Mc3r* mice is independent of improvements in adiposity. These results suggest that actions involving NKX2.1(+ve);MC3R(+ve) neurons in the hypothalamus are sufficient to restore “normal” activity of NAG neurons. This could indicate a developmental role in which NAG neurons fail to develop normal responses to altered signals of metabolic state in the absence of MC3Rs. Alternatively, MC3Rs in the mature hypothalamus may exert an active “gating” function; determining whether rescuing MC3Rs in the adult mouse restores responses of NAG neurons to metabolic cues could address this question.

MC3Rs expressed in the limbic system may regulate feeding-related motivational responses. MC3Rs are expressed in dopamine transporter (DAT) (+ve) and (−ve) neurons in the VTA, with female *Mc3r*-deficient mice exhibiting lower dopamine and altered sucrose consumption and taste preferences (Lippert et al., 2014). Operant conditioning experiments suggest increased food-related motivational responses associated with hypocaloric diets are attenuated in *Mc3r*-deficient mice. Rescuing *Mc3r* transcription in DAT(+ve) neurons in the VTA improved motivational responses (Mavrikaki et al., 2016) without restoring binge-feeding observed following the prolonged inter-meal interval. Compulsive behavioral responses to consume large meals in situations of negative energy balance may thus require MC3R activation in additional brain areas, and not only in the limbic system. A caveat to interpreting these studies is that they only used male mice; sex differences in the functions of MC3Rs in regulating feeding-related reward pathways exist (Lippert et al., 2014). Further studies using LoxTB*MC3R* mice to investigate the role of MC3Rs expressed in the VTA of females in regulate sucrose consumption and taste preferences are clearly needed.

## Melanocortin-3 receptors: role in metabolic control

Early experiments examining hypophyseal and autonomic outputs from the CNS controlling metabolism by melanocortins suggested no requirement for MC3R signaling. Acute stimulation of sympathetic activity by melanotan-II (MTII), an α–MSH analog, requires functional MC4Rs (Haynes et al., 1999). The regulation of energy expenditure by melanocortins is mediated by MC4Rs expressed by cholinergic sympathetic pre-ganglionic neurons; glucose control involves MC4Rs expressed on both sympathetic and parasympathic cholinergic pre-ganglionic neurons (Rossi et al., 2011; Sohn et al., 2013; Berglund et al., 2014).

Similar to appetite control, the role of MC3Rs in metabolic homeostasis may also be contextual. We have reported two studies suggesting that MC3R signaling has a role in maintaining metabolic homeostasis and insulin sensitivity. The first study examined metabolic responses of *Mc3r*−*/*− mice subjected to the hypocaloric conditioning protocol used to induce FAA (Sutton et al., 2010; Begriche et al., 2011b; Girardet et al., 2014b). *Mc3r*−*/*− mice fed a single low-fat/high carbohydrate meal at 24 h intervals exhibited rhythms in hyperinsulinemia and insulin-regulated genes involved in lipid synthesis in the liver that peaked around meal presentation. This outcome suggests partial insulin resistance, with hepatic insulin sensitivity retained while other tissues (presumably skeletal muscle) are insulin resistant. While rhythms in insulin and glucose *ad libitum* fed *Mc3r*−*/*− mice were normal, this result might be misleading. Fasting insulin, fasting glucose and glucose tolerance are normal in muscle-specific insulin receptor knockout mice (MIRKO) (Bruning et al., 1998). Moreover, muscle insulin resistance redistributes nutrients to adipose tissue, increasing adiposity (Kim et al., 2000). It is therefore possible that *Mc3r*−*/*− mice are insulin resistant in skeletal muscle; showing this is the case requires more sensitive methodologies for measuring glucose metabolism. It might also be informative to examine entrainment of metabolic control to hypocaloric conditioning in MIRKO.

The second study involved rescuing *Mc3r* expression in steroidogenic factor-1 (SF1, also known as NR5A1) expressing neurons in the VMH (Begriche et al., 2011a). Early studies using in situ hybridization revealed the VMH as a site of dense *Mc3r* expression (Roselli-Rehfuss et al., 1993). Mice expressing Cre in VMH SF1(+ve) neurons (SF1-Cre) have been used to manipulate the expression of genes expressing hormone and growth factor receptors (leptin, insulin, estrogen, BDNF), second messenger signaling pathways and transcription factors involved in metabolic control (Kim et al., 2011; Klöckener et al., 2011; Orozco-Solis et al., 2015, 2016; Berger et al., 2016). VMH SF1(+ve) regulate glucose metabolism, regulate glucose production (Tong et al., 2007; Garfield et al., 2014; Meek et al., 2016). SF1(+ve) neurons are thus involved in the defense of body weight and metabolic control.

**Figure 2:**
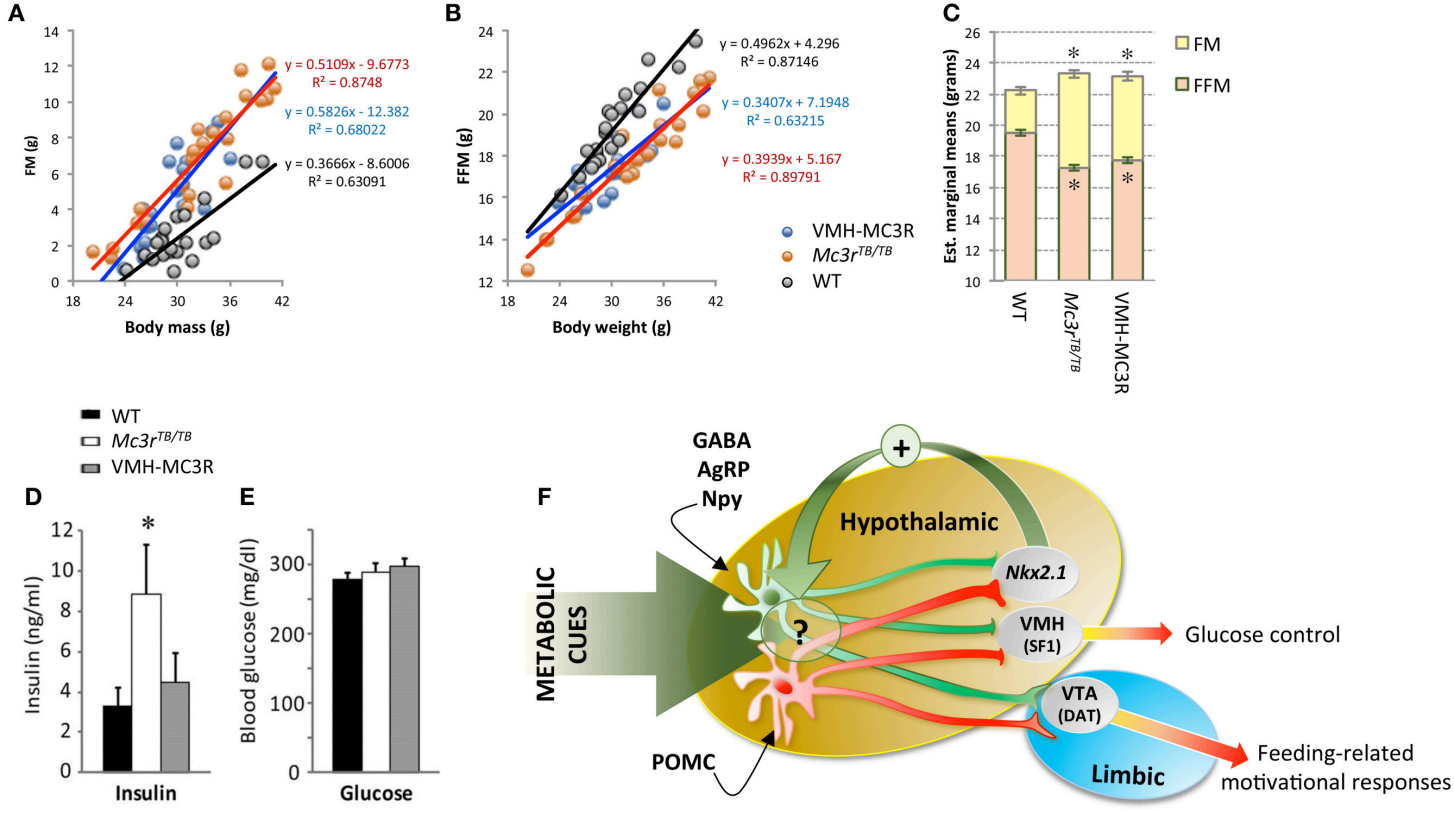
**Improved insulin sensitivity in VMH-MC3R mice is independent of reduced adiposity (A–E)** and model describing the physiological roles of MC3Rs in the brain **(F)**. A regression approach plotting fat mass (FM, **A**) and fat-free mass (FFM, **B**) determined using NMR demonstrates the nutrient partitioning phenotype. As body mass increases, gains in FM are proportionately increased while gains in FFM are proportionately reduced in homozygous carriers of the “lox-stop-stop” suppressed *Mc3r* gene (*Mc3r*^*TB*/*TB*^). Analysis of body composition using analysis of covariance (ANCOVA) using total body mass as a covariate indicates the predicted reduction of FFM and increased FM in *Mc3r*^*TB*/*TB*^ mice. In *Mc3r*^*TB*/*TB*^ mice where transcription in the ventromedial hypothalamus has been rescued (VMH-MC3R), the slope of association between FM and FFM as a function of body mass is similar to *Mc3r*^*TB*/*TB*^ mice **(A,B)**; estimated marginal means derived from ANCOVA are also similar in VMH-MC3R and *Mc3r*^*TB*/*TB*^ mice **(C)**. Fasting insulins are significantly increased in *Mc3r*^*TB*/*TB*^ mice compared to controls and VMH-MC3R mice (**D**, ^*^p < 0.05), with no difference in blood glucose **(E)**. Model describing functional distribution of MC3Rs in the CNS suggested by studies using Cre transgenes to restore transcription in the VMH (SF1-Cre), VTA (DAT-Cre) and hypothalamus (Nkx2.1-Cre). MC3Rs expressed on SF1(+ve) neurons in the VMH are sufficient to improve metabolic control, while MC3Rs expressed in dopamine transporter (DAT) (+ve) neurons in the VTA restore feeding-related motivational responses during situations of caloric insufficiency. MC3Rs expressed in Nkx2.1-Cre(+ve) neurons are sufficient to restore normal responses of NAG (GABA/AgRP/Npy) neurons to signals of negative energy state, and for expression of food anticipatory activity and binge-feeding responses during situations of negative balance. While some Nkx2.1(+ve);MC3R(+ve) neurons reside in the hypothalamus, their specific location and identity remain unknown. In addition, while the actions of Nkx2.1(+ve);MC3R(+ve) neurons appears to be critical for the normal regulation of NAG neurons in response to metabolic cues, the underlying mechanism remains unknown.

We crossed SF1-Cre and LoxTB*Mc3r* mice, rescuing Mc3r expression in the VMH (VMH-MC3R). Analysis of body composition (fat mass, fat-free mass) using a regression approach (Packard and Boardman, 1988; Allison et al., 1995) indicates that the nutrient partitioning phenotype is not rescued (Figures 2A–C). The expression of FAA was also not rescued (Begriche et al., 2011a). However, significant improvements in fasting insulin were observed in the absence of changes in fasting glucose (Figures 2D,E). In addition, changes in hepatic gene expression suggesting increased fatty acid flux were also partially reversed (Begriche et al., 2011a). The dissociation of the effects of MC3R on obesity from altered metabolic control suggests that MC3Rs expressed by SF1(+ve) neurons in the VMH are involved in metabolic control.

Regulation of peripheral metabolism by MC3Rs may not be “acute,” in that stimulation of MC3Rs in the absence of MC4Rs does not produce rapid changes. However, reduced fasting insulin in *Mc4r*−*/*− mice treated with an MSH analog for 14d suggests MC4R-independent effects on insulin sensitivity (Kumar et al., 2009). Whether this response involved MC3Rs expressed in the VMH or elsewhere has not been determined.

## Summary and future perspectives

The functions of neural MC3Rs received little attention after the publication of the phenotypes of *Mc3r*−*/*− mice in 2000. However, MC3Rs in the CNS regulate feeding-related motivational behaviors and glucose homeostasis. Both phenotypes appear to be context-dependent, increasing in prevalence with negative energy balance. Hypothalamic MC3R signaling maintains sensitivity of the nutrient-sensing networks in the hypothalamus to signals of metabolic condition (Figure 2E). In humans, *MC3R* polymorphisms have been associated with reduced interest in food (Lee et al., 2007; Obregon et al., 2010; Aris et al., 2015). Given the contextual nature of the feeding phenotype in mice, studies examining feeding behavior in humans with *MC3R* polymorphisms should consider energy balance in their experimental design. Finally, while making progress in identifying MC3Rs involved in appetite control, the location(s) of MC3Rs affecting nutrient partitioning remains unclear.

## Author contributions

AB prepared the first manuscript draft. CG, MM, JT, HM, DM, and SF reviewed and edited the manuscript.

## Funding

Some of the work cited in the article was supported by grants from the National Institutes of Health (DK073189) to AB. AB also thanks the support of the Pennington Biomedical Research Foundation, Clinical Nutrition Center Grant P30 DK072476 (“Nutritional Programming: Environmental and Molecular Interactions”), The Scripps Florida Fund and financial support from Saint Louis University.

### Conflict of interest statement

The authors declare that the research was conducted in the absence of any commercial or financial relationships that could be construed as a potential conflict of interest.
